# RETRACTION: Schiff Base Metal Derivatives Enhance the Expression of HSP70 and Suppress BAX Proteins in Prevention of Acute Gastric Lesion

**DOI:** 10.1155/bmri/9821953

**Published:** 2026-06-11

**Authors:** BioMed Research International

RETRACTION: S. Golbabapour, N. S. Gwaram, M. M. J. Al‐Obaidi, A. F. Soleimani, H. M. Ali, and N. A. Majid, “Schiff Base Metal Derivatives Enhance the Expression of HSP70 and Suppress BAX Proteins in Prevention of Acute Gastric Lesion,” *BioMed Research International*, 2013, 703626, 10.1155/2013/703626

The above article, published online on 05 November 2013 in Wiley Online Library (wileyonlinelibrary.com), has been retracted by agreement between the journal Editor‐in‐Chief, Yoel Klug, and John Wiley & Sons Ltd. The retraction has been agreed due to concerns with Figure 2, initially highlighted on PubPeer [1]. Specifically:

• Figure 2q appears to be identical to Figure 7e in [2], albeit rotated 180 degrees.
• Figure 2q appears to share overlapping regions with Figure 3b in [3].

The authors could not provide the original images, and after assessment by the editorial board the article is retracted.

Mazen M. Jamil Al‐Obaidi agrees to the retraction. No response was received from the remaining authors.
